# Viable Newcastle Disease Vaccine Strains in a Pharmaceutical Dump

**DOI:** 10.3201/eid1312.070715

**Published:** 2007-12

**Authors:** Antonella Amendola, Silvia Bianchi, Marta Canuti, Alessandra Zappa, Giovanna Zanoni, Raffaella Koncan, Elisabetta Tanzi, Giuseppe Cornaglia, Alessandro Remo Zanetti, Giuseppe Tridente

**Affiliations:** * University of Milan, Milan, Italy; † University of Verona, Verona, Italy

**Keywords:** Buried pharmaceutical waste, biological risk, NDV vaccine, waste yard, reclaim, dispatch

## Abstract

To assess the viability of discarded and buried vaccine strains, we examined vaccines that had been buried for >20 years in an industrial waste dump in the city of Milan, Italy. Viability results showed potential biological risk associated with uncontrolled burial of pharmaceutical industry waste, including some live vaccines.

During most of the 20th century, biopharmacologic products, including vaccines, prophylactic serum, blood flasks, and animal-origin waste, were buried <1.5 m deep in the 12,000-m^2^ waste dump of a pharmaceutical research institute in Milan (Istituto Sieroterapico Milanese [ISM]). ISM was founded in 1886 but bankrupt by 1994. This dump area was recently reclaimed after nearly a decade of abandonment.

Some of the material with potential biological risk, such as animal carcasses, has been decomposing in direct contact with the soil. Other material, including vaccines against human and animal diseases such as rabies, poliomyelitis, anthrax, and Newcastle disease (ND), were recovered in hermetically sealed vials, so their contents might have been totally or partially preserved. Our aim in this preliminary study was to assess the viability of the unearthed vaccines.

## The Study

The reclamation procedure was performed under strict safety conditions. All operations were conducted under biocontainment tents that had air exchangers and extractors with filters. Full personal protective equipment, including biohazard suits, gas masks, and gauntlets, was provided to all workers. The amount of biopharmacologic waste removed was impressive (35,764 tons). In particular, large quantities of ND vaccines were recovered, in liquid and in lyophilized form; the estimated net weight of biological material was 20–25 kg.

Four vaccine types against ND (2 in liquid and 2 in lyophilized form) were unearthed from the dump. A review of documentation found that all 4 types had been produced by ISM from 1975 through 1988. Because the exact composition of the vaccines was unknown, the vial contents were subjected to viability tests in culture and to molecular characterization assays. The viability evaluations were performed by inoculation of the vial contents onto a confluent monolayer of Vero cells (African green monkey kidney), followed by observation of the cell culture for 7 days and assessment of any cytopathic effect (*1*). Each assay was performed twice. The positive control was a commercially available ND vaccine (Izovac; IZO S.p.A, Brescia, Italy), containing >10^6^ 50% embryo infectious dose live attenuated viruses.

**Figure 1:**
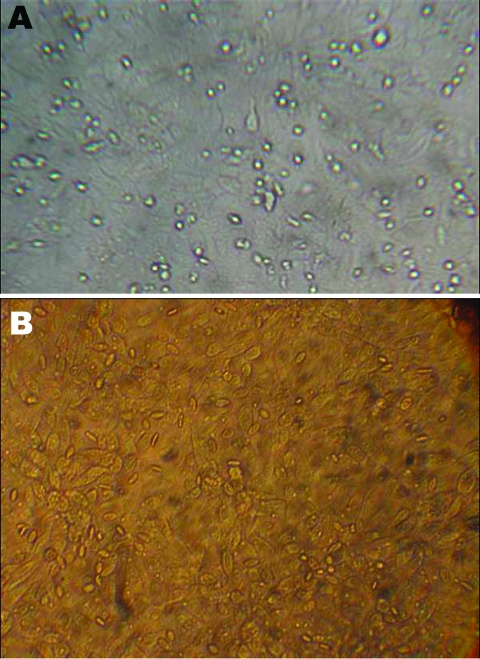
A) Cytopathic effect of lyophilized Newcastle disease virus (NDV) vaccine strains on Vero cells. B) Hemagglutination test. Presence of tear-shaped erythrocytes confirms the specificity of the cytopathic effect of NDV on Vero cells.

As a result of bacterial contamination, no virus could be isolated from the 2 types of liquid vaccine. However, virus was isolated from the 2 lyophilized ND vaccine strains and caused cytopathic effect, which was further confirmed by hemoadsorbing assay (Figure 1). These lyophilized strains, which showed vitality in Vero cells, were then propagated in embryonated chicken eggs, the preferred substrate for ND virus (NDV) growth. Briefly, samples were inoculated into the allantoic cavity of 10-day-old embryonated chicken eggs and further incubated at 36°C for 5 days. Eggs were subsequently chilled to 4°C, and the allantoic fluid was tested for hemagglutination (HA) activity to confirm the presence of viral replicaion (*2*). A 512-fold increase of HA titer was observed after infection (i.e., from 4 HA units in the whole, resuspended vaccine to 2.048 HA units in the allantoic fluid).

**Figure 2:**
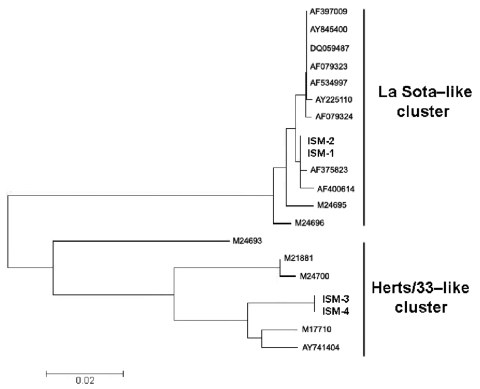
Phylogenetic analysis of Newcastle disease virus (NDV) vaccine strains unearthed from Istituto Sieroterapico Milanese (ISM), showing the phylogenetic placement of ISM-1 (EU082818), ISM-2 (EU082819), ISM-3 (EU082820), and ISM-4 (EU082818) based on partial F gene necleotide sequences. Sequences determined in this study are in **boldface.** ISM-1 and ISM-2 belong to La Sota–like cluster; ISM-3 and ISM-4 belong to Herts/33-like cluster. Sequence alignment was achieved with ClustalX version 1.81 (ftp://ftp.ebi.ac.uk/pub/software/unix/clustalx), with sequences from other NDV strains retrieved from GenBank (accession nos. indicated). The phylogenetic study was conducted by using MEGA version 3.1 (www.megasoftware.net). The phylogenetic tree was constructed with the neighbor-joining method.

An additional part of the original vial content of the unearthed vaccines was used for molecular characterization. Viral RNA extracted from all 4 vaccine types (liquid and lyophilized) was subjected to sequencing of the genomic region, including the cleavage site, which is the determinant of virulence for NDV strains (*3*,*4*). For this purpose, vial content was subjected to genic amplification of a 615-bp fragment (*5*) of the F gene, which encodes for the fusion protein. The nucleotide sequence obtained was aligned with sequences in online databases by using ClustalX software (ftp://ftp.ebi.ac.uk/pub/software/unix/clustalx). The amino acid sequence was then predicted with BioEdit software (www.mbio.ncsu.edu/bioedit/bioedit.html), and a detailed analysis was performed on the cleavage site (Table). The phylogenetic tree of the F gene fragment (Figure 2) provided the vaccine strain’s classification. The lyophilized samples contained La Sota–like strains classified as lentogenic strains. The liquid samples contained genomic sequences of the cleavage site characteristic of Herts/33-like strains (velogenic strains).

**Table:** Comparison of Newcastle disease virus sequences

Classification*	Amino acid sequence
Lentogenic	^112^(G/E)(R/K)Q(G/E)RL^117^
Velogenic	^112^(R/K)RQ(R/K)RF^117^
Strains†	Nucleotide/amino acid sequence
ISM-1, ISM-2	GGG AGA CAG GGG CGC CTT ^112^G R Q G R L^117^
ISM-3, ISM-4	AGG AGA CAG AGA CGG TTT ^112^R R Q R R F^117^

*Amino acid sequences at the F protein cleavage site of lentogenic and velogenic strains of Newcastle disease virus. †Nucleotide/amino acid sequences of the samples recovered from a dump at Istituto Sieroterapico Milanese (ISM), Milan, Italy, and analyzed in this study. ISM-1 and ISM-2 (GenBank accession nos. EU082818 and EU082819, respectively) were recovered in lyophilized form; ISM-3 and ISM-4 (GenBank accession nos. EU082820 and EU082821, respectively) were recovered in liquid form. The cleavage site sequences of ISM-1 and ISM-2 are typical of lentogenic strains; the cleavage site sequences of ISM-3 and ISM-4 are typical of velogenic strains.

## Conclusions

Vast areas that were once used as uncontrolled dumps for pharmaceutical industry waste still exist in many countries. One example is the waste dump of the ISM, which was 1 of the leading Italian companies in the field of research and development of vaccines against the infectious diseases most prevalent at the time, including diphtheria, smallpox, tetanus, anthrax, rabies, and poliomyelitis.

Because of this unusual line of production, the reclamation activities of the ISM area took into consideration the potential biological risks caused by the buried and unprocessed waste. This first attempt in Italy to reclaim such an area yielded large quantities of biological material, including well-preserved vaccines and by-products of their manufacturing processes. Because data about the manufacturing and disposal procedures used at the time are missing, the recovery of such biological materials raises concerns about persistent biological activity. Moreover, veterinary vaccines, especially those in lyophilized form, may represent a relevant biological risk because they are often prepared with strains that have been attenuated for the target animals but not for humans.

This preliminary study considered the residual pathogenic potential of ND vaccines. ND is a viral infection of poultry, caused by an avian paramyxovirus serotype 1 (*6*), which may cause human disease and may pose a hazard to exposed workers (*7*,*8*). ND infections usually cause unilateral or bilateral reddening and edema of the eyelids, excessive lacrimation, conjunctivitis, and subconjunctival hemorrhage. ND infections are usually transient with no corneal involvement (*9*); however, severe complications leading to lasting vision impairment have been described (*10*).

The viability data of lyophilized strains of live NDV vaccines, conserved in hermetically sealed vials and buried for >20 years, showed that the strains had persisting replication ability in Vero cells and in embryonated chicken eggs. This residual vitality implies that manipulation of discarded vaccines may involve risk for infection. Molecular characterization of the F gene classified the lyophilized vaccines as derived from lentogenic strains. On the contrary, the cleavage site of the liquid vaccines contained genomic sequences characteristic of velogenic strains. That the liquid vaccine originally consisted of inactivated NDV or of velogenic strains (used in the past) attenuated by several passages in culture systems is possible.

This study indicates the existence of biological risk deriving from the uncontrolled burial of vaccines and their by-products and underlines the absence of worldwide-accepted criteria defining the extent and persistence of biological risk–related biopharmacologic waste materials. The results of the study support the need to plan and perform rational reclamation operations in abandoned biopharmaceutical waste areas, implementing biocontainment strategies and personal and environmental safety measures. These measures are particularly necessary in those situations in which the buried material could contain highly infectious and pathogenic agents such as pox and anthrax, which were largely used by leading vaccine producers in the past century.

In conclusion, further studies are needed to fill knowledge gaps regarding disposed biological material. Such studies are warranted to evaluate the extent and the persistence of the infectious risk brought about by buried vaccines.
